# Predictive value of different scoring systems for critically ill patients with hospital acquired pneumonia

**DOI:** 10.1186/2197-425X-3-S1-A345

**Published:** 2015-10-01

**Authors:** A Abouelela, T Al-Badawy, M Abdel Gawad

**Affiliations:** Critical Care Medicine, Alexandria University, Alexandria, Egypt

## Introduction

Hospital-acquired pneumonia (HAP) and ventilator-associated pneumonia (VAP) are important causes of morbidity and mortality despite improved antimicrobial therapy, supportive care, and prevention. General risk factors for developing HAP include age older than 70 years, serious comorbidities, malnutrition, impaired consciousness, prolonged hospitalization and COPD. The availability of valid criteria for defining severe pneumonia would provide a more reliable basis for improving patients risk assessment.

## Objectives

Is to assess the prognostic value of 7 different scores: Pneumonia Severity Index (PSI),CURB65, Modified ATS rule, infectious Diseases Society of America/American Thoracic Society Consensus Guidelines (IDSA/ATS), SMART COP, Simplified SMART-COP (SMART CO) and SOAR) in assessing the severity of HAP and outcome of patients.

## Methods

This is a prospective Cohort study performed on a sixty patients admitted to critical care medicine department of Alexandria University Hospital in Egypt over 12 months. All patients were diagnosed as HAP.Calculation of the mentioned 7 scores was done once diagnosis of HAP was confirmed.

## Results

The Area Under the Curve was highest in SMART-cop (AUC:= 0.820) followed by the SMART-CO score (AUC: = 0.807) and PSI score(AUC: = 0.806). All the previous scores SMART-cop score at Cutoff value ≥ 2, SMRT-Co Score at Cutoff value ≥ 2, Modified ATS score at Cutoff value ≥ 0.5 and PSI (pneumonia severity index) at Cutoff value ≥ 3. have the highest sensitivity (sensitivity 100% for each) in predicting 28-day mortality. regarding Specificity, SMART-cop score is the most specific one (Specificity= 93%) in predicting 28-day mortality followed by Modified ATS score (Specificity= 90%). regarding the duration of Mechanical Ventilation it was found that SMART-cop (R = 0.824, p = 0.0001) followed by IDSA/ATS scores (R= 0.787, p = 0.0001) had the highest correlation in predicting duration of Mechanical Ventilation in critically ill patient with VAP as a higher SMART-cop and IDSA/ATS score reflect that the pneumonia was complicated with septic shock and respiratory failure.

## Conclusions

SMART - cop score is the most sensitive score in predicting 28 day mortality in the studied patient followed by SMART - co and PSI score). SMART-cop score is the most specific one (Specificity= 93%) in followed by Modified ATS score (Specificity= 90%).
